# Lung Cancer Screening With Low-Dose CT: Its Effect on Smoking Behavior

**DOI:** 10.6004/jadpro.2013.4.6.3

**Published:** 2013-11-01

**Authors:** Meaghan McEntee Gomez, Geri LoBiondo-Wood

**Affiliations:** From the University of Texas MD Anderson Cancer Center, Houston, Texas

## Abstract

Lung cancer screening provides an opportunity for smoking cessation interventions. A review of the literature found that smokers who participated in lung cancer screening had a higher smoking cessation rate compared with smokers in the general population. However, the randomized controlled trials included in the review did not identify any difference in smoking cessation rates between the individuals who had a CT scan to screen for lung cancer and unscreened control groups. Multiple studies observed participants for lengths of time ranging from 1 to 36 months and concluded that individuals who received abnormal CT results had a higher smoking cessation rate compared with participants with normal CT results. A single study that observed participants for 6 years initially found similar increased cessation rates among those with abnormal CT results, but at the conclusion of the study the difference in cessation rates had dissipated. Lung cancer screening produces a teachable moment when individuals may be more receptive to smoking cessation interventions. Advanced practitioners should take an active role in promoting smoking cessation interventions and fostering this teachable moment created by lung cancer screening.

Lung cancer is the leading cause of cancer death in the United States, with an estimated 228,190 new cases and 159,480 deaths predicted in 2013 (Siegel, Naishadham, & Jemal, 2013). Most lung cancers are diagnosed in later stages: 57% of individuals with lung cancer have distant metastases at diagnosis, 22% present with spread to regional lymph nodes, and 15% are diagnosed with only localized disease (Howlader et al., 2013). Individuals diagnosed at earlier stages have a higher probability of cure and a higher 5-year survival rate compared with those diagnosed at later stages. The average 5-year survival rate for lung cancer patients is 15.6% (Howlader et al., 2013). Individuals diagnosed with localized disease have a 52% 5-year survival rate compared with 3.6% for patients who present with metastatic disease. These statistics highlight the need for an effective screening tool to detect lung cancer at its early stages.

Computed tomography (CT) screening for lung cancer can potentially diagnose patients at an early disease stage. Although previous trials had not shown a mortality benefit from CT screening (Doria-Rose et al., 2009; Marcus et al., 2006; Melamed et al., 1984), in August 2011 results from the National Lung Screening Trial (NLST) demonstrated that low-dose CT screening was correlated with a reduction in lung cancer mortality compared with chest radiography screening. In this trial, more than 53,000 individuals at high risk for lung cancer had three annual screenings with CT scans or chest radiography. The participants were enrolled in the trial from August 2002 to August 2004 and observed through December 2009. Eligible participants had a smoking history of 30 pack-years and if had quit smoking, had done so in the past 15 years, and were between 55 and 74 years old.

 The NLST found a relative reduction of 20.3% in lung cancer mortality and a 7% reduction in all-cause mortality for those randomized to undergo low-dose CT screening compared with those screened with chest radiography (Aberle et al., 2011). The results from the NLST have the potential to change clinical practice for individuals at high risk for lung cancer by providing earlier diagnosis, thereby increasing the chance of long-term survival.

## Background and Significance

Lung cancer is the most preventable type of cancer; 80% to 90% of lung neoplasms are attributed to smoking (Wingo et al., 1999). In the United States, 21% of people over the age of 18 currently use tobacco (Pleis, Ward, & Lucas, 2010). Smoking cessation is an effective method of reducing lung cancer mortality (Peto et al., 2000). With the recently reported mortality benefit for people at high risk for lung cancer as demonstrated by the NLST, lung cancer screening could become standard practice. Smoking cessation in addition to lung cancer screening could further decrease the rate of lung cancer–associated mortality. The purpose of this review is to evaluate the literature on CT screening for lung cancer and its effect on smoking behavior. This information has numerous potential implications for health-care interventions aimed at facilitating smoking cessation for patients participating in lung cancer screening.

## Methods and Relevant Literature

The databases searched were MEDLINE (Ovid), CINAHL (Cumulative Index to Nursing and Allied Health Literature), and the Cochrane Library. Search terms used were "tomography" or "x-ray computed" or "screening" and "lung neoplasms" or "lung cancer" or "lung carcinoma" and "smoking cessation." Searches were limited to the English language, but the publication years were not limited. The searches yielded 113 articles. Animal studies, editorials, commentaries, abstracts, and articles pertaining to screening by sputum or chest radiography alone were excluded. Also excluded were studies that were not relevant to CT screening for lung cancer and its effect on smoking behavior. One study was omitted because it measured intended smoking cessation rather than actual cessation. This review comprises 11 studies. All eligible studies were retrieved, and their references were examined for further relevant publications. Of the 11 studies included in the review, 3 were randomized controlled trials (RCTs) and 8 were nonexperimental in design. The Table provides an overview of the included studies.

**Table.**
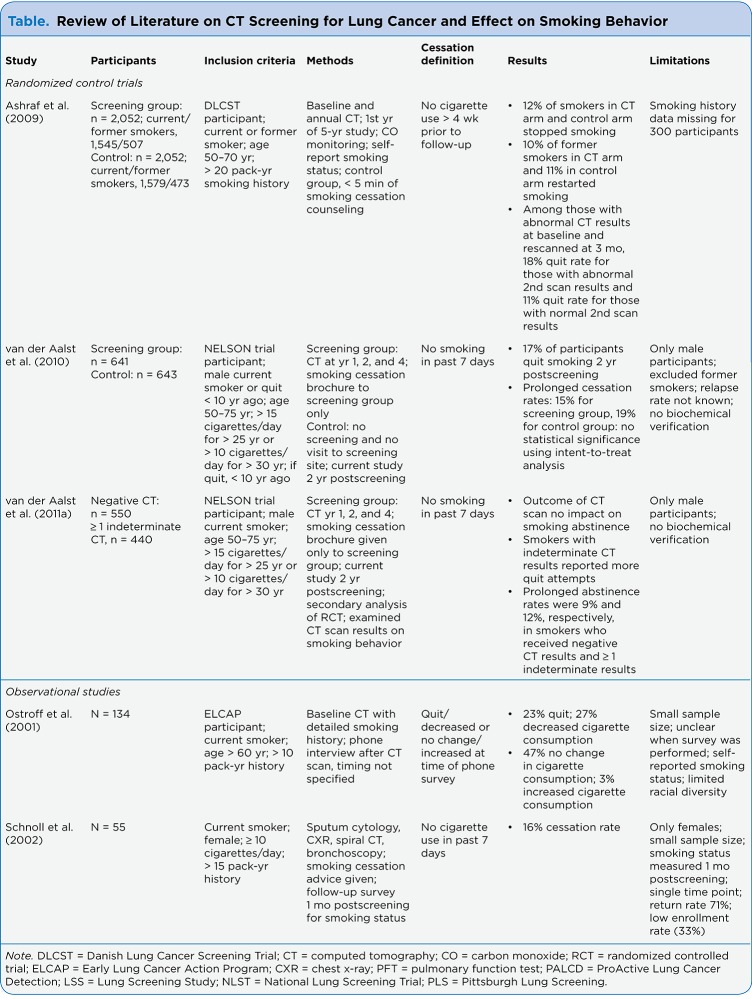
Table. Review of Literature on CT Screening for Lung Cancer and Effect on Smoking Behavior

**Table.**
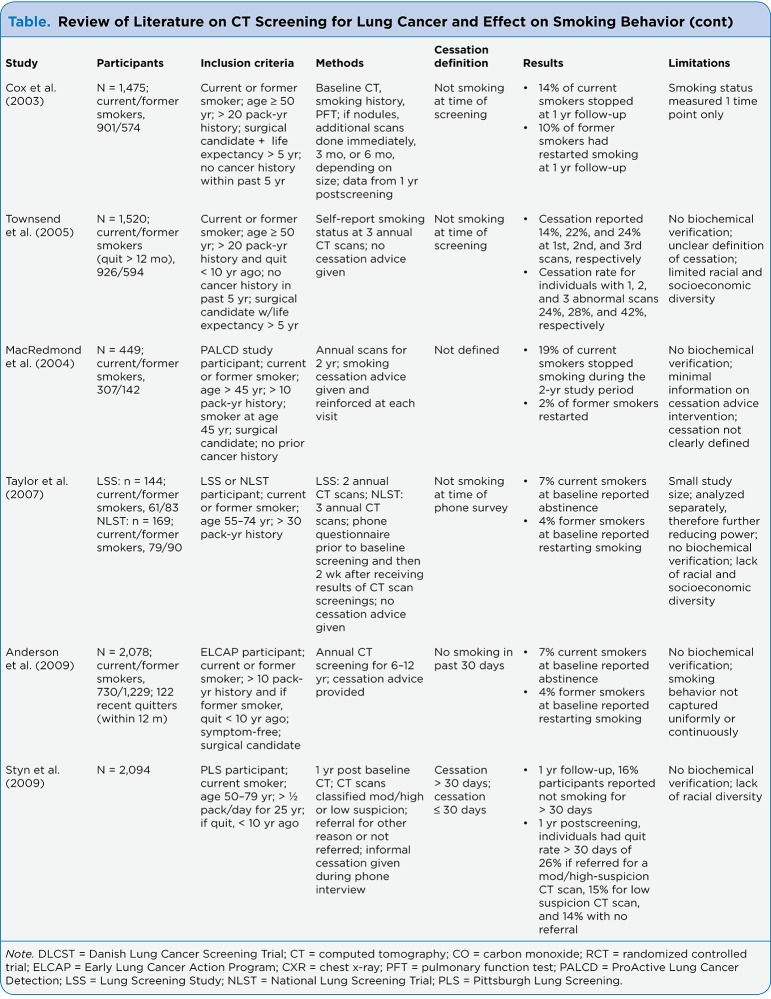
Table. Review of Literature on CT Screening for Lung Cancer and Effect on Smoking Behavior (cont)

## Literature Synthesis

**HYPOTHESIZED EFFECTS OF SCREENING ON SMOKING BEHAVIOR**

A number of nonrandomized screening programs have examined the potential negative effect of CT lung cancer screening on smoking behavior (Cox et al., 2003; Ostroff, Buckshee, Mancuso, Yankelevitz, & Henschke, 2001; Townsend et al., 2005). It is hypothesized that CT screening could create a "green light" effect—a false sense of security that results in a lower motivation to quit and thereby encourages individuals to continue to smoke (Ostroff et al., 2001). However, multiple studies have not found the green light effect to be present, as there has been no evidence of increased cigarette consumption after participating in screening (Anderson et al., 2009; Ashraf et al., 2009; Cox et al., 2003; Schnoll et al., 2002; van der Aalst, van den Bergh, Willemsen, de Koning, & van Klaveren, 2010). The other proposed hypothesis is that screening may offer a "teachable moment" that could lead to increased smoking cessation rates. The term teachable moment is used to explain health events that motivate individuals to adopt risk-reducing health behaviors (McBride, Emmons, & Lipkus, 2003); the term has frequently been used in analyses of smoking cessation interventions.

**POPULATION SAMPLE FACTORS**

To fully comprehend the multifaceted process of quitting smoking, it is necessary to examine motivation and interest level of smokers regarding screening and smoking cessation. Two studies demonstrated that a high percentage of smokers (62% and 77%) were interested in participating in lung cancer screening (Hahn, Rayens, Hopenhayn, & Christian, 2006; Schnoll et al., 2003). Taylor et al. (2007) explored the individuals’ reasons for participation in a lung cancer screening trial; common motivations reported included psychological factors, such as wanting peace of mind about lung cancer and being worried about lung cancer, and altruistic motivations, such as wanting to make a contribution to medical science. A small number of responses were reasons related to personal awareness, such as screening recommended by others, family history of lung cancer, and symptoms of lung cancer.

Another study found that interest in lung cancer screening was associated with perception of cancer risk and knowledge of asymptomatic illness (Schnoll et al., 2003). It has also been shown that current smokers who participated in lung cancer screening were interested in smoking cessation (Ostroff et al., 2001; Taylor et al., 2007; Townsend et al., 2005), specifically nicotine replacement therapy and cessation counseling interventions (Taylor et al., 2007). Therefore, the data suggest that smokers are willing to have lung cancer screening and that individuals who are screened want additional assistance and information on smoking cessation.

**SMOKING BEHAVIOR**

The three RCTs and one observational trial suggest that participants in lung cancer screening did not exhibit a change in smoking behavior (Anderson et al., 2009; Ashraf et al., 2009; van der Aalst et al., 2010; van der Aalst, van Klaveren, van den Bergh, Willemsen, & de Koning, 2011b). The RCTs’ unscreened control groups had quit rates similar to those of the groups who had CT screening (Ashraf et al., 2009; van der Aalst et al., 2010; van der Aalst et al., 2011b).

Ashraf et al. (2009) randomly assigned participants to undergo either annual CT screening (n = 2,052) or no screening (n = 2,052). The participants had a smoking history of at least 20 pack-years and received minimal (< 5 minutes) smoking cessation counseling. Individuals were classified as ex-smokers if they had not smoked in 4 weeks prior to the baseline screening visit. At the 1-year follow-up visit, quit rates were 11.9% in the CT screening group and 11.8% in the control group. Baseline predictors for quitting smoking at the 1-year follow-up included worse pulmonary function tests, lower average number of cigarettes smoked per day since starting smoking, lower Fagerström Nicotine Dependence Questionnaire scores, and higher motivation to quit (Ashraf et al., 2009).

The NELSON trial randomly assigned 11,181 men into screening (n = 5,438) and control (n = 5,451) arms, excluding females and unknown gender (n = 292). Participants in the screening arm had a baseline CT scan and responded to a questionnaire that assessed demographic factors, smoking behavior variables, and attitudes toward smoking cessation. More data were collected on these variables 2 years later (van der Aalst et al., 2010; van der Aalst et al., 2011b). van der Aalst et al. examined a subgroup of NELSON participants who were current smokers, defined as smoking in past the 7 days, randomly selected from the trial’s screening (n = 641) and control (n = 643) groups (van der Aalst et al., 2010). The researchers found that 16.6% of trial participants stopped smoking, which is appreciably higher than the cessation rate among smokers in the general population. The screening group had a lower prolonged cessation rate (14.5%) than the control group (19.1%), but an intent-to-treat analysis found no statistical difference.

Another article that reported no change in smoking behavior related to screening was an observational trial by Anderson et al. (2009), who examined a subpopulation of participants from the Early Lung Cancer Action Program. This study included current smokers (n = 730), long-term former smokers (n = 1,229), and recent quitters (n = 122); participants were observed for up to 12 years. Anderson et al. (2009) found there was no long-term smoking abstinence or increased relapse over the minimum 6-year time frame participants were observed.

The results of the observational studies demonstrate that people who participated in lung cancer screening had high smoking cessation rates: between 7% and 23% (Cox et al., 2003; MacRedmond et al., 2004; Ostroff et al., 2001; Schnoll et al., 2002; Styn et al., 2009; Taylor et al., 2007). These statistics are noteworthy, especially compared with the quit rate for the general population, which is 2% to 3% (Stead, Bergson, & Lancaster, 2008). In one study, 87% of participants who had stopped smoking named participation in the trial as the major influence in their motivation for smoking cessation (Ostroff et al., 2001).

**EFFECT OF CT SCAN RESULTS**

A few studies examined the effect of positive and negative CT scan results on the smoking cessation rates of participants in lung cancer screening. Multiple studies indicate that abnormal CT results encourage smoking cessation (Ashraf et al., 2009; Styn et al., 2009; Townsend et al., 2005). Townsend and colleagues (2005) conducted a longitudinal study that examined current (n = 926) and former smokers (n = 594) who had three annual CT scans to screen for lung cancer. During the 3-year follow-up, abstinence from smoking was also associated with older age, worse baseline pulmonary function tests, and abnormal CT scan results the previous year, requiring closer follow-up. Smoking abstinence was reported by 41.9% of individuals who had abnormal CT scan results for all three screenings, 28.0% of those with two abnormal screening results, 24.2% of those with one abnormal screening result, and 19.8% of those with no abnormal screening results (Townsend et al., 2005).

Another study determined that participants who were referred to a physician because of moderately to highly suspicious CT results had an 18.8% higher rate of quitting for more than 30 days compared with those who had normal scan results and were not referred to a physician (Styn et al., 2009). Taylor et al. (2007) separately analyzed the effect of CT results on smoking behavior among participants from two different screening trials (the NLST and the Lung Screening Study) and had conflicting findings. The Lung Screening Study participants were more ready to quit if they had received abnormal results, but among the NLST participants, the screening results had no effect on cessation.

Anderson and colleagues (2009) initially came to a similar conclusion as the previous investigators. Individuals who had negative CT scan outcomes were 28% less likely to report not having smoked within the past 30 days compared with those who had a positive CT result that required further evaluation. But after 6 years, the statistical significance between the cessation rates of the two groups had dissipated. This implies that positive CT scans may initially increase rates of smoking cessation but do not improve rates of prolonged smoking abstinence, defined as not consuming cigarettes for more than 1 year (Anderson et al., 2009).

van der Aalst and colleagues (2011a) found comparable outcomes when examining the effect of CT results on smoking behavior in an additional subset population of the NELSON trial. In this study, two randomly selected groups of male smokers with negative (n = 550) or indeterminate (n = 440) CT scan results had their smoking status examined 2 years after their baseline CT scan. The data suggest that there was no statistically significant difference in smoking cessation rates between the two groups. However, individuals who had only received negative results had made fewer quit attempts than had participants with indeterminate CT scan results.

## Summary of Results

In summary, both RCTs and observational studies suggest that trial participants in both screening and control groups were more inclined to stop smoking as compared with smokers in the general population. The RCTs revealed no significant difference between individuals screened with CT and individuals who were not screened. The RCTs helped clarify whether an individual’s cessation was associated with the CT screening or the act of participating in a lung cancer screening program. Most studies concluded that individuals who had abnormal CT results that required additional follow-up had higher cessation rates than did those with negative CT results.

**STUDY LIMITATIONS**

The majority of studies included in this review were nonexperimental (Anderson et al., 2009; Cox et al., 2003; MacRedmond et al., 2004; Ostroff et al., 2001; Schnoll et al., 2002; Styn et al., 2009; Taylor et al., 2007; Townsend et al., 2005), while three were RCTs (Ashraf et al., 2009; van der Aalst et al., 2010; van der Aalst et al., 2011b). Although both types of studies demonstrated the relationship between participation in a smoking cessation trial and smoking cessation, the RCTs more clearly demonstrated the effect of CT results. In the RCTs, the quit rate for both the control and experimental groups was significantly higher than the average cessation rate among smokers in the general population. If the trials had not had control groups, the increased smoking cessation rate compared to the general population could have been attributed to CT screening (Ashraf et al., 2009).

There is a concern that the study populations are not randomly selected because participation in lung cancer screening trials was voluntary; people who take part may be more motivated to quit than other smokers (van der Aalst et al., 2010). In addition, smokers expressed more interest in lung cancer screening if they were preparing to quit or thinking about quitting smoking than those who were not motivated to quit, actively quitting, or maintaining abstinence (Hahn et al., 2006).

Another issue was that many of the reviewed studies had minimal ethnic diversity in their populations; the majority of participants were white, with other groups vastly underrepresented. In the articles in which ethnicity/race was listed, 83% to 99% of the participants were white (Anderson et al., 2009; Cox et al., 2003; Ostroff et al., 2001; Styn et al., 2009; Taylor et al., 2007; Townsend et al., 2005). In 2009, 22.1% of current smokers in the United States above the age of 18 years were White and non-Hispanic and 21.3% were black (Dube et al., 2010). The majority of the articles had equivalent gender proportions, but two of the studies had only male participants (van der Aalst et al., 2010; van der Aalst et al., 2011b) and one study included only female smokers (Schnoll et al., 2002). It is essential that the study population be representative of the general population so that cultural, racial, and gender differences can be appreciated and taken into consideration when creating smoking cessation interventions to accompany lung cancer screening programs.

An additional limitation was that cessation was defined in various ways in the different studies: not smoking at the time of follow-up (Taylor et al., 2007), in the past 7 days (Cox et al., 2003; van der Aalst et al., 2010), in the past 30 days (Styn et al., 2009), or in the past 4 weeks (Ashraf et al., 2009). One study further defined point abstinence as having refrained from smoking cigarettes for 30 days, cumulative point abstinence as having not smoked in more than 30 days but less than 1 year, and long-term abstinence as having refrained from smoking for 1 year (Anderson et al., 2009). In another study, cessation was described as not smoking in the past 7 days or having had fewer than 5 cigarettes since the quit date (van der Aalst et al., 2011b). The variety of definitions of cessation can make it difficult to compare results from studies, and it is understandable how the different studies could have a variety of outcomes.

The length of time researchers observed participants after lung cancer screening and acquired information about their smoking status varied immensely among studies. One study observed participants for a minimum of 6 years (Anderson et al., 2009), and a handful of studies tracked patients for 1 to 2 months (Schnoll et al., 2002; Taylor et al., 2007), while the majority of articles reported researchers observing patients from 1 to 3 years (Ashraf et al., 2009; Cox et al., 2003; MacRedmond et al., 2004; Styn et al., 2009; Townsend et al., 2005; van der Aalst et al., 2010; van der Aalst et al., 2011b). The variety in follow-up periods could result in different conclusions.

Multiple articles relied on participant self-reported smoking status without biochemical confirmation (Anderson et al., 2009; MacRedmond et al., 2004; Ostroff et al., 2001; Townsend et al., 2005; van der Aalst et al., 2010; van der Aalst et al., 2011b), and a few studies used biochemical monitoring to confirm smoking status (Ashraf et al., 2009; Cox et al., 2003; Styn et al., 2009). Self-reported smoking status could introduce a social response bias; however, Studts et al. (2006) examined the validity of self-reported smoking status in lung cancer screening participants and found a total miscalculation rate of only 7%. As this study had a sensitivity of 91% and a specificity of 95%, the risk of a social response bias is limited.

## Clinical Implications

As participation in lung cancer screening is associated with smoking cessation, there are additional elements of lung cancer screening trials that could encourage smoking cessation by the creation of a teachable moment. Smokers often name physician advice as a major factor motivating cessation (Kviz, Clark, Hope, & Davis, 1997). A short smoking cessation recommendation by a health-care provider can increase quit rates by 1% to 3%, with a small added benefit from more intensive interventions (Stead, Bergson, & Lancaster, 2008). Repeat CT scans for lung cancer screening allow multiple opportunities to promote cessation interventions and provide follow-up support to recent quitters. In addition, recurring scans help to establish a rapport between the health care worker and patient. Therefore, lung cancer screening offers an opportunity for advanced practitioners to promote smoking cessation by seizing the teachable moment.

Receiving abnormal results or a possible cancer diagnosis during lung cancer screening may provide a teachable moment that could help motivate individuals to quit smoking. McBride, Emmons, and Lipkus (2003) proposed three domains that help identify whether an event is significant enough to develop into a teachable moment. A significant event does one of the following: "(1) increases perceptions of personal risk and outcome expectancies, (2) prompts strong affective or emotional responses, and (3) redefines self-concept or social role" (McBride et al., 2003, p. 156).

Accessing these three domains could help advanced practitioners make the most of the potential teachable moment by having smoking cessation discussions with patients during lung cancer screening. Individuals who take part in lung cancer screening can experience increased personal risk perception and outcome expectancies. Screened individuals can have altered cancer-related risk perceptions, which can help motivate smokers to quit (Cox et al., 2003; Ostroff et al., 2001; Townsend et al., 2005), and it is thought that increased cancer risk perception can result in behavior change (Kreuter & Strecher, 1995). Individuals who believe that continuing to smoke will result in negative health outcomes are the most likely to quit smoking (McBride et al., 2003). If people participating in screening have a vivid and explicit experience of risk, such as a possible cancer diagnosis, this may result in a change in smoking behavior. The fear of the scan uncovering possible lung cancer creates the potential for an intense emotional response when a patient is participating in lung cancer screening. Also, a possible cancer diagnosis could intensify the belief that smoking is not acceptable or is unsuited to the participant’s role and/or obligations, increasing the motivation to quit.

As lung cancer screening becomes more standardized (i.e., interval of screening, stratification by risk, cost-effectiveness) and guidelines are created, smoking cessation must be an integral part of lung cancer screening programs. A multidisciplinary approach using pharmacotherapy, counseling, and motivation from screening results will be fundamental to these cessation programs. Advanced practitioners must take an active role in educating patients about screening and smoking cessation. In one study, 77% of responders were unaware that spiral CT was being used to screen for lung cancer (Schnoll et al., 2003). It is imperative that advanced practitioners raise awareness about CT screening for lung cancer. It is equally essential to identify and refer individuals who qualify for lung cancer screening. Advanced practitioners in oncology should play a critical role in promoting smoking cessation for patients within lung cancer screening programs and capitalizing on the related teachable moments.

Many unknowns remain concerning smoking cessation related to CT screening for lung cancer, including the timing and type of cessation interventions that are most effective, which indicates a need for further research. Some data show improved cessation rates for cessation treatment interventions given prior to the screening test compared with interventions given after the screening results were disclosed (Ferketich et al., 2011). In addition, specific cessation methods continue to be debated. Individualized lung cancer risk and giving personalized smoking cessation information may not be more effective than providing participants with general information (Clark et al., 2004; van der Aalst et al., 2011b). More research is needed to help evaluate the most effective methods of smoking cessation with lung cancer screening. Additional RCTs and studies with longer follow-up are essential to help to evaluate long-term smoking cessation and to help decrease the number of issues related to population and study design.

## Conclusions

In summary, smokers who participate in lung cancer screening are more likely to stop smoking than smokers in the general population. Randomized controlled trials found no statistically significant difference between individuals who were screened with CT and individuals who were not screened. The information gained from this review should help motivate advanced practitioners to promote smoking cessation interventions, as lung cancer screening participants have about a three to four times higher chance of successfully quitting smoking than smokers in the general population. It should be emphasized to health-care providers that lung cancer screening provides a teachable moment that can be used to encourage smoking cessation and reduce future morbidity in this subset of patients.
